# Urotensin II Exerts Pressor Effects By Stimulating Renin And Aldosterone Synthase Gene Expression

**DOI:** 10.1038/s41598-017-12613-y

**Published:** 2017-10-24

**Authors:** Brasilina Caroccia, Mirko Menegolo, Teresa M. Seccia, Lucia Petrelli, Michele Antonello, Alice Limena, Andrea Porzionato, Raffaele De Caro, Marko Poglitsch, Gian Paolo Rossi

**Affiliations:** 1 Clinica dell ‘Ipertensione Arteriosa, Department of Medicine-DIMED, University of Padua, Padua, Italy; 2 Vascular and Endovascular surgery, Department of Cardiac Thoracic and Vascular Sciences, University of Padua, Padua, Italy; 3 Human Anatomy, Department of Molecular Medicine, University of Padua, Padua, Italy; 4 Attoquant Diagnostics, Vienna, Austria

## Abstract

We investigated the *in vivo* pressor effects of the potent vasoconstrictor Urotensin II (UII). We randomized normotensive Sprague-Dawley rats into 4 groups that received a 7-day UII infusion (cases) or vehicle (controls). Group 1 received normal sodium intake; Group 2 underwent unilateral nephrectomy and salt loading; Group 3 received spironolactone, besides unilateral nephrectomy and salt loading; Group 4 only received spironolactone. UII raised BP transiently after a lag phase of 12-36 hours in Group 1, and progressively over the week in Group 2. Spironolactone did not affect blood pressure, but abolished both pressor effects of UII in Group 3, and left blood pressure unaffected in Group 4. UII increased by 7-fold the renal expression of renin in Group 2, increased aldosterone synthase expression in the adrenocortical zona glomerulosa, and prevented the blunting of renin expression induced by high salt. UII raises BP transiently when sodium intake and renal function are normal, but progressively in salt-loaded uninephrectomized rats. Moreover, it increases aldosterone synthase and counteracts the suppression of renin induced by salt loading. This novel action of UII in the regulation of renin and aldosterone synthesis could play a role in several clinical conditions where UII levels are up-regulated.

## Introduction

Urotensin II (UII), a somatostatin-like cyclic 11-aminoacid peptide originally identified in the caudal neuro-secretory system of teleost fish, is a potent vasoactive peptide that concentration-dependently contracts vascular smooth muscle *in vitro* ^1–3^. It circulates at low plasma concentrations and activates a specific G-protein coupled receptor, thus sharing functional similarities with other potent vasoactive peptides, as endothelin 1 and angiotensin II^4^^,^^5^. Of note, the plasma levels of UII were found to be increased in several conditions, including chronic kidney disease, haemodialysis^6^, and diabetes mellitus^7^. Moreover, UII and Ang II appear to act synergistically in contracting isolated rat aortae with^8^ and without intact endothelium^9^, suggesting an interaction and/or a cross-talk between UII and the renin-angiotensin-aldosterone system at the effector site. Because of these features, it was suggested that UII can play a key pathophysiological role in the cardiovascular system^10–13^, but this remains contentious thus far, as, notwithstanding the clear-cut *in vitro* vasoconstrictor effect, acute *in vivo* studies gave conflicting results. In monkey infusions of UII increased total peripheral resistance and left ventricular (LV) end-diastolic pressure, but lowered mean blood pressure (BP), carotid blood flow, and cardiac output^14^. In normotensive rats UII caused vasodilation, dose-dependently decreased mean BP, LV systolic pressure and dP/dt, leading to fatal circulatory collapse^15^^,^^16^. Moreover, even though systemic BP was unaffected, a chronic infusion of UII (300 µmol/Kg/h) markedly decreased myocardial contractility and increased LV end-diastolic pressure, possibly because of coronary vasoconstriction and/or collagen deposition in the LV^17^. This intriguing discrepancy between the *in vitro* vasoconstriction and the *in vivo* fall of BP remains to be mechanistically explained. We hypothesize that these different results could be due to the different experiment settings involving acute vs chronic studies and different timing and mode of blood pressure measurements. Accordingly, only an *in vivo* study using telemetry to measure BP could clarify the pictures.

UII and its receptor (UT-R) are expressed in the human and rat adrenocortical zona glomerulosa^18^^,^^19^, and we previously showed that a chronic infusion of UII into normotensive rats increased the expression of the aldosterone synthase (Cyp11b2) gene, e.g. the key enzyme required for the conversion of 11-deoxicortisol to aldosterone^20^. These findings led to hypothesize that UII could raise BP not only through direct vasoconstriction, but also by enhancing aldosterone secretion. If this were the case, administration of exogenous UII would be expected to increase BP only transiently, considering that in animals with normal renal function acutely induced hyperaldosteronism is rapidly followed by an “escape” of BP and sodium retention. In fact, when infused into animals with a normal renal function aldosterone initially raises BP through salt and water retention, but after few days BP normalizes due to a blunted synthesis of endogenous aldosterone, deriving from renin suppression and increased natriuretic peptides, and the onset of a water and natriuretic response mediated by the latter peptides. This initial pressor effect might have been missed by measuring BP at single discrete time points, and could be ascertained only with a continuous beat-to-beat invasive radio-telemetry BP monitoring. Hence, we set up this study to verify this hypothesis and to further explore the effects of chronically infused UII on the renin-angiotensin-aldosterone system.

## Results

**Figure 1:**
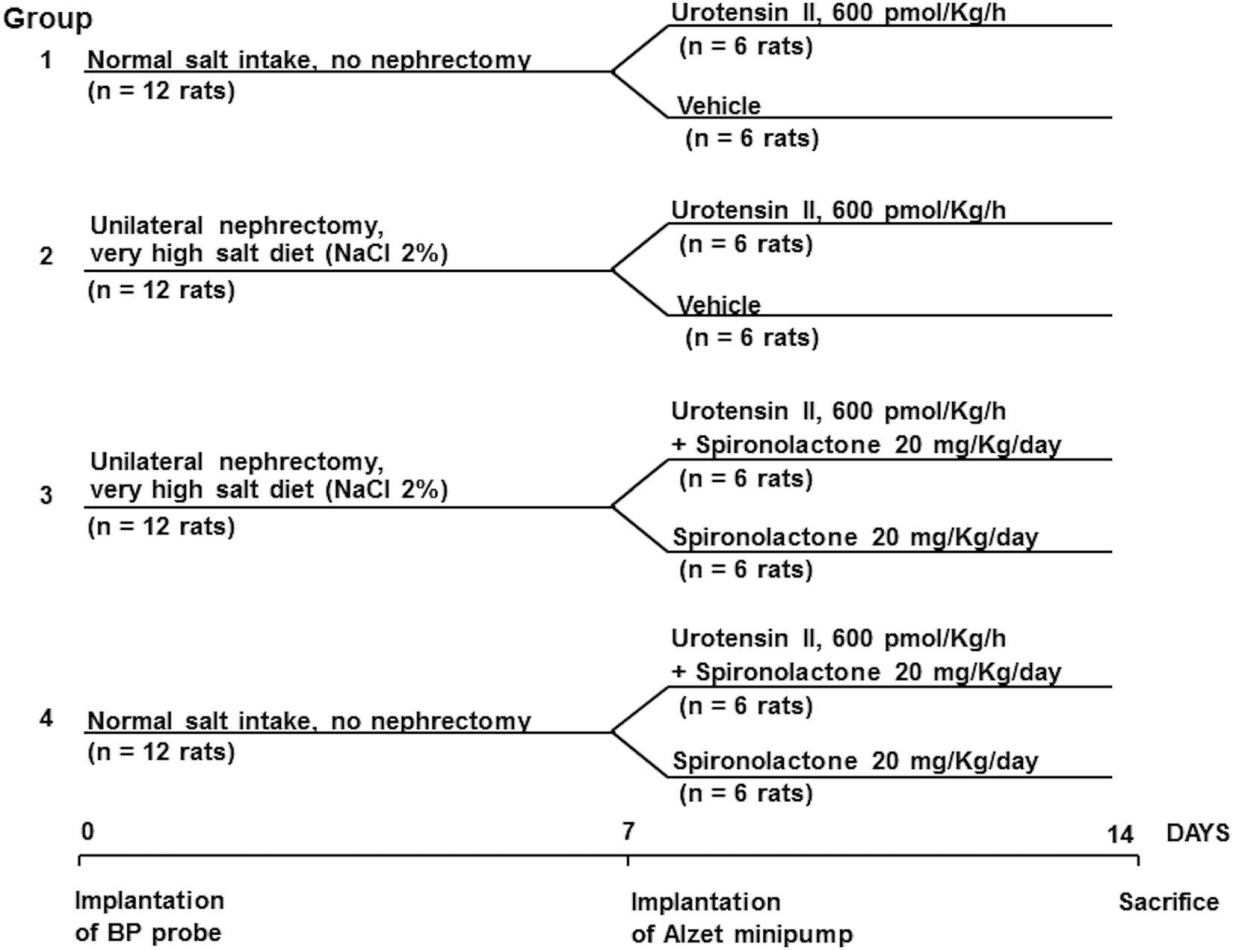
Study design. 15 weeks-old male Sprague-Dawley rats were divided into 4 groups of 12 animals divided into 6 cases that received a 7-days infusion of urotensin II and 6 controls receiving vehicle. Npx = unilateral nephrectomy; Na = sodium; UII = urotensin II, 600 pmol/kg/h; Spiro = spironolactone, 20 mg/kg/day. See text for details.

Male BP- and weight-matched Sprague-Dawley rats (Charles River Laboratories, Wilmington, MA) at 15 weeks of age were divided into 4 groups of 12 animals each, split into 6 cases and 6 controls (Fig. 1). In Group 1 cases and controls received a 7-days infusion of either UII, or vehicle, respectively; details are reported in methods section. In Group 2 the animals receiving the same infusion of UII or vehicle were also submitted to high (2% NaCl) sodium intake and to unilateral nephrectomy. In Group 3, besides nephrectomy and high sodium intake, rats also received the mineralocorticoid receptor (MR) antagonist spironolactone that was co-administered with UII. In Group 4 rats received UII and spironolactone infusion.

Table 1 shows the raw and body weight-indexed LV weight recorded in each group. As compared to controls, rats receiving UII in Group 1 and 2 showed a significantly higher LV and LV/BW ratio.

**Table 1:** Left ventricle (LV) weight and left ventricle over body weight (LV/BW) in cases and controls of each group.

Group	LV (mg)			LV/BW (mg/g)		
	Cases	Controls	p	Cases	Controls	p
Group 1 (UII)	930 ± 20	860 ± 30	0.01	2.44 ± 0.11	2.27 ± 0.01	0.02
Group 2 (UII + UNX + HS 2% diet)	680 ± 19*	470 ± 40*	0.02	2.85 ± 0.44	2.36 ± 0.18	0.03
Group 3 (SPIRO + UNX + HS 2% diet)	620 ± 11*	500 ± 70*	ns	2.48 ± 0.21	2.49 ± 0.33	ns
Group 4 (SPIRO)	740 ± 50**	830 ± 70**	ns	2.05 ± 0.12^**^	2.22 ± 0.31	ns

UNX: nephrectomy; SPIRO: spironolactone; HS 2% diet: high sodium 2% diet.

Mean (±SD); *p < 0.001, vs group 1; **p < 0.01, vs group 1.

**Figure 2:**
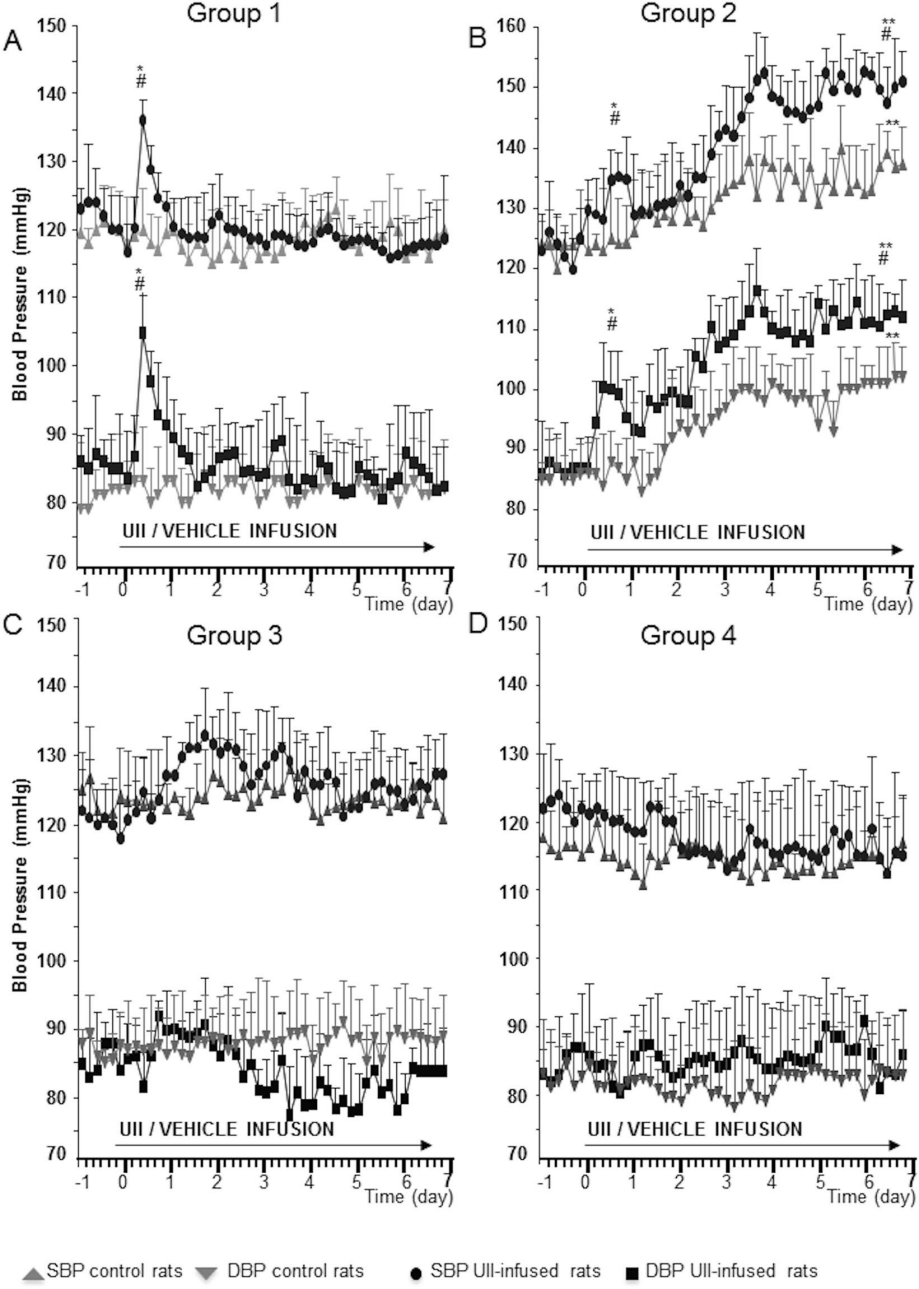
BP profiles. Systolic and diastolic BP changes at beat-to-beat radio-telemetry recording in rats of Group 1 receiving UII (panel A, normal sodium diet), in unilaterally nephrectomized rats of Group 2 receiving UII and high (2%) NaCl diet (panel B, High Sodium + Uninephrectomy), in rats of Group 3 similarly treated but receiving also spironolactone (panel C, High Sodium + Uninephrectomy + Spironolactone), and in rats of Group 4 that received UII and spironolactone (panel D, Normal Sodium + Spironolactone). Mean ± SD; n = 6 *per* group. *p < 0.001 day 1 vs day 0; **p < 0.001 day 7 vs day 0; ^#^p < 0.01 UII infused rats vs vehicle infused rats.

In Group 1 the rats receiving UII for 7 days showed a transient BP increase that occurred after a lag-phase of about 12/36 hours (Fig. 2, panel A, black symbols). This pressor response never occurred in the control rats receiving the vehicle in an identical fashion (Fig. 2, panel A, gray symbols).

In Group 2 the rats showed good general conditions, as the 2% NaCl intake in drinking water allowed enough fluid intake to avoid dehydration. After 12-24 hours of the infusion, the cases showed a progressive increase of BP values (Fig. 2, panel B, black symbols cases, gray symbols controls). Some BP increase occurred also in Group 2 control rats that were uninephrectomized and exposed to the high salt diet; however, this pressor effect was significantly blunted, as compared to the similarly treated rats that received UII, and attained significance from baseline only at the last time point.

Neither the early transient nor the late pressor response to UII were seen in Group 3, which differed from Group 2 only for the concomitant administration of the MR antagonist spironolactone that was started at the same time of UII via osmotic mini-pumps (Fig. 2, panel C, black symbols cases, gray symbols controls). Thus, BP did not show any increase in rats treated with spironolactone.

In Group 4 spironolactone administration, which was well tolerated during the entire study period, caused no significant changes of BP from baseline values (Fig. 2, panel D, black symbols cases, gray symbols controls).

### Renin and UT-R expression in the kidney

Double immuno-staining evidenced renin (red) in the juxta-glomerular (JG) cells of the vascular pole of glomeruli and UT-R (brown) localization in the vascular wall, mainly in endothelium, of glomerular arterioles (Supplemental Fig. 1, panel A). Immunohistochemistry showed prominent staining of the UT-R of pre- and post-glomerular arterioles in the renal cortex, (Supplemental Fig. 1, panel A) of the different groups, thus supporting the contention that UII can act at this level.

### Renal effect of UII

**Figure 3:**
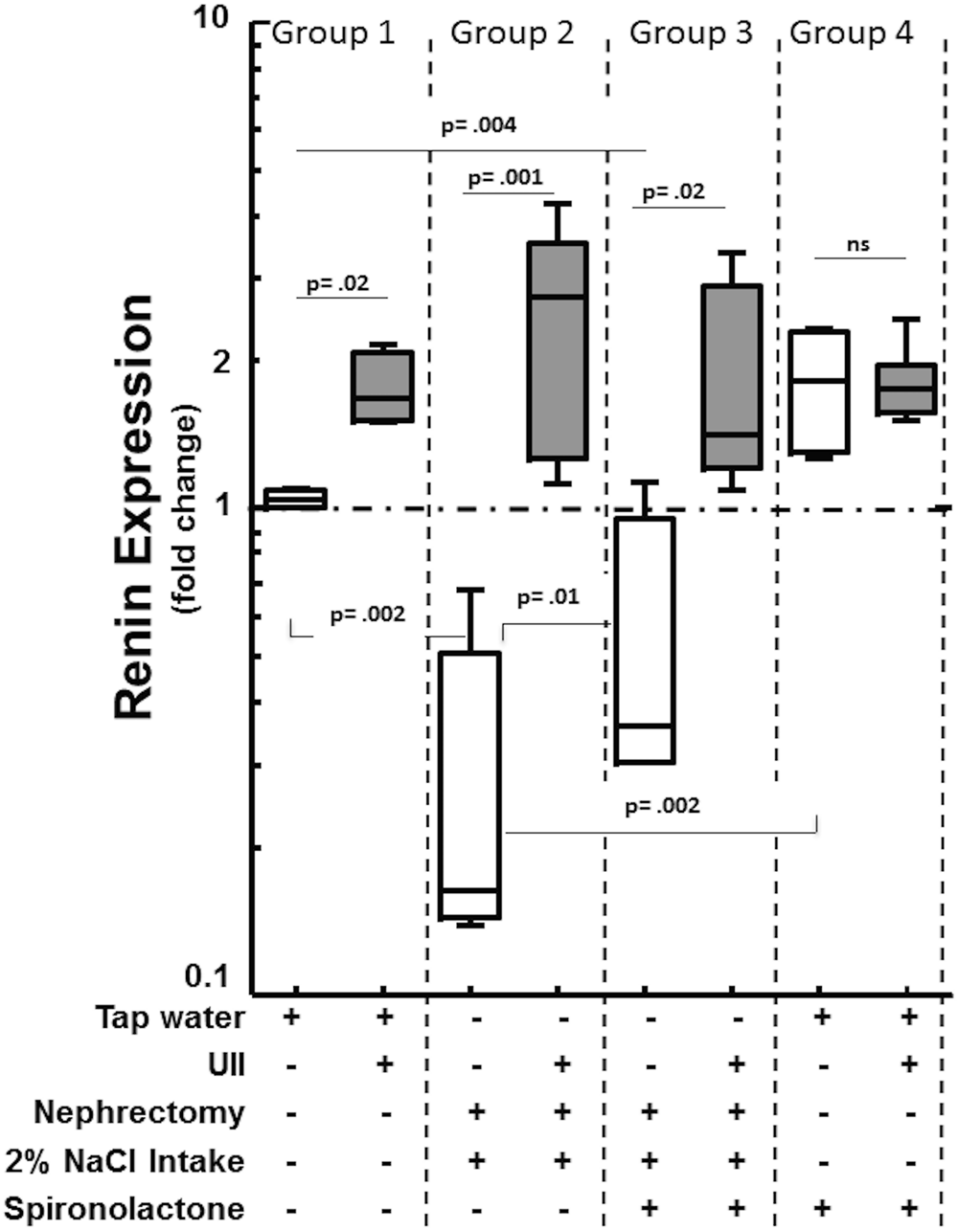
UII infusion increased renin gene expression in the kidney. Box and whisker plot showing the fold changes (in log_10_ scale) of renin gene expression in the kidney (measured by ∆∆CT method using PBGD mRNA as a reference), relative to vehicle-infused (control) rats. UII induced a marked increase in rats with normal salt intake and renal function (Group 1), as well as in uninephrectomized rats undergoing 2% sodium loading (Groups 2-3). Renin expression was blunted in uninephrectomized rats undergoing 2% sodium loading that did not receive UII (controls). Please note that spironolactone increased renin in Group 3 and in Group 4 as compared to uninephrectomized and salt loaded animals. Median and 95% CI, n = 6 *per* group, one-way ANOVA and post-hoc Bonferroni’s test.

In renal sections of Group 1 controls immunostaining for renin occurred in juxta-glomerular (JG) cells (Supplemental Fig. 1, panel B; Supplemental Fig. 2, panel A). After UII infusion both the number of renin-positive cells and the intensity of staining increased in Group 1 cases (Supplemental Fig. 1, panel C; Supplemental Fig. 2, panel B). In these rats UII induced a prominent (2-fold) increase of renin gene expression in the renal cortex (Fig. 3). In Group 2 and 3 controls, the number of renin-positive cells and the amount of renin in each cell was less evident than in Group 1 controls (Supplemental Fig. 1, panel D; Supplemental Fig. 2, panel C and panel E). The renin gene expression was also blunted in uninephrectomized rats undergoing 2% NaCl loading (Fig. 3). By contrast, it was markedly activated by UII: not only renin immunostaining was evident in renal JG cells and arterioles of cases, but extended along almost the entire afferent arteriole and the distal tubules (Supplemental Fig. 1, panel E; Supplemental Fig. 2, panel D and panel F). In Group 4 spironolactone increased renin at the gene and protein level, in both controls and cases; UII co-treatment did not further enhance this increase (Fig. 3; Supplemental Fig. 2, panel G and panel H).

### Renin and angiotensin peptides in the kidney

We measured renin and angiotensin peptides in the renal tissue based on the premise that the changes occurring at the tissue level could provide more accurate information on the UII effects. The results of these measurements, carried out blindly to the experimental groups at Attoquant in Vienna, showed that in the animals of Group 2 that received UII there was a consistent increase of renin, Ang 1-10, Ang 1-8, Ang 2-8, Ang 1-7 (Supplemental Table 2).

### Effect of UII on Cyp11b2

**Figure 4:**
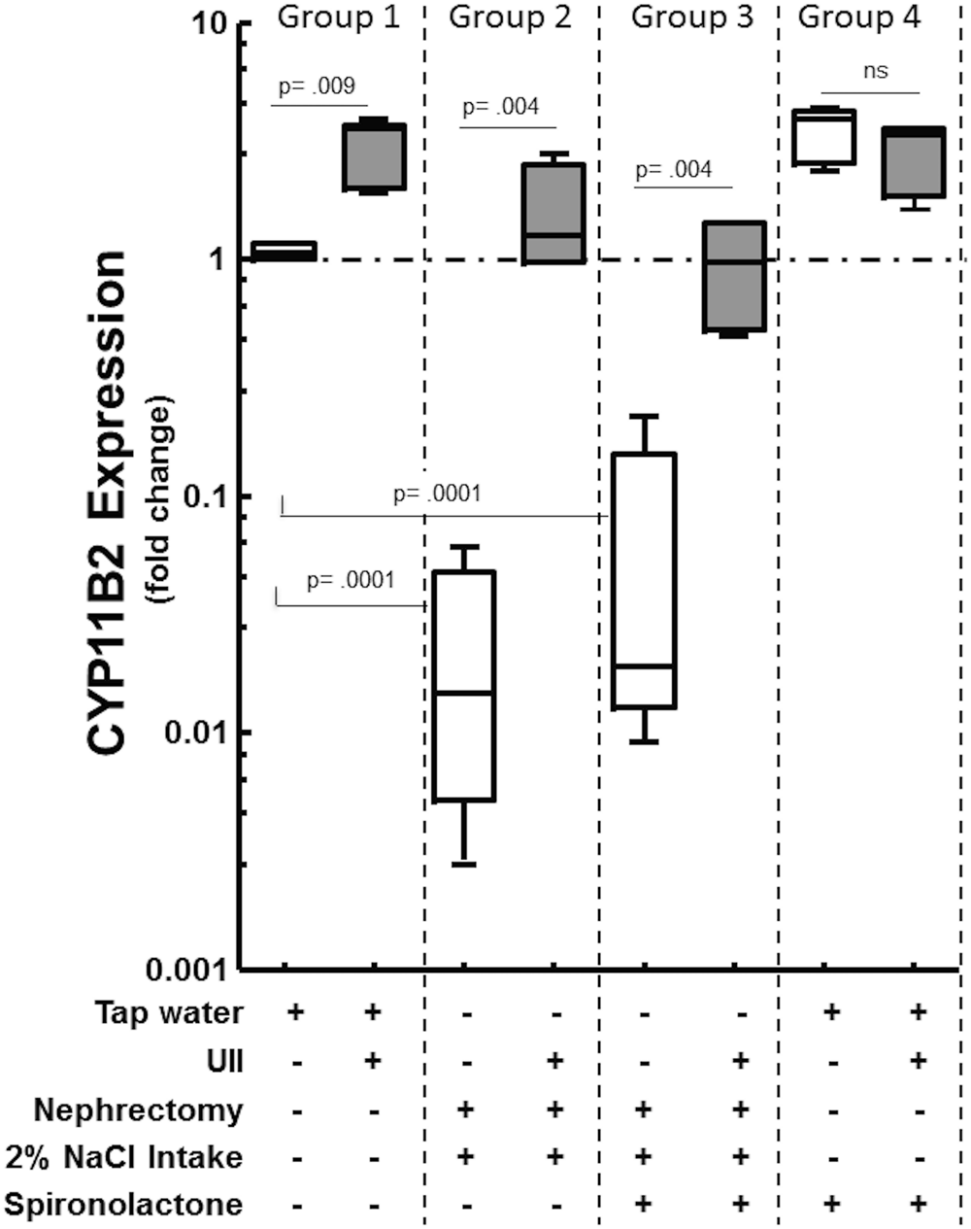
UII infusion markedly increased Cyp11b2 gene expression in the zona glomerulosa. Box and whisker plot showing the fold change relative to measurements in vehicle-infused (control) rats in Cyp11b2 gene expression in the adrenocortical zona glomerulosa. UII increased Cyp11b2 expression in rats with normal salt intake and normal renal function (Group 1), and in uni-nephrectomized rats undergoing 2% sodium loading (Group 2-3). Cyp11b2 gene expression was blunted in uni-nephrectomized rats undergoing 2% sodium loading. Median (5-95 percentile), n = 6 rats *per* group, one-way ANOVA and post-hoc Bonferroni’s test.

Controls in Group 1 showed only weak CYP11B2 immunostaining in the zona glomerulosa (Supplemental Fig. 3, panel A). In the same Group cases (UII-treated rats) infusion of UII induced a prominent increase in zona glomerulosa width as evidenced by an increase in the number of cell layers expressing aldosterone synthase (Supplemental Fig. 3, panel B), along with 3-fold increase of Cyp11b2 gene expression compared to controls (vehicle treated rats) (Fig. 4). In control rats of Group 2 and 3 immunostaining for CYP11B2 was markedly blunted by high-salt diet, although some positive CYP11B2-expressing cells remained detectable (Supplemental Fig. 3, panel C and panel E). In cases of Group 2 and 3 UII infusion caused a prominent increase in the number of CYP11B2-stained cells in the zona glomerulosa (Supplemental Fig. 3, panel D and panel F). The Cyp11b2 gene expression decreased markedly in the adrenocortical zona glomerulosa of sodium loaded uninephrectomized rats (Fig. 4). However, similarly to what observed for renin, in cases of Group 2 and 3, UII prevented the salt-loading-induced decrease of this gene (Fig. 4). In Group 4 spironolactone increased gene and protein expression of aldosterone synthase, but UII co-treatment did not elicit any further increase (Fig. 4; Supplemental Fig. 3, panel G and panel H).

### Effect of UII on aldosterone secretion

**Figure 5:**
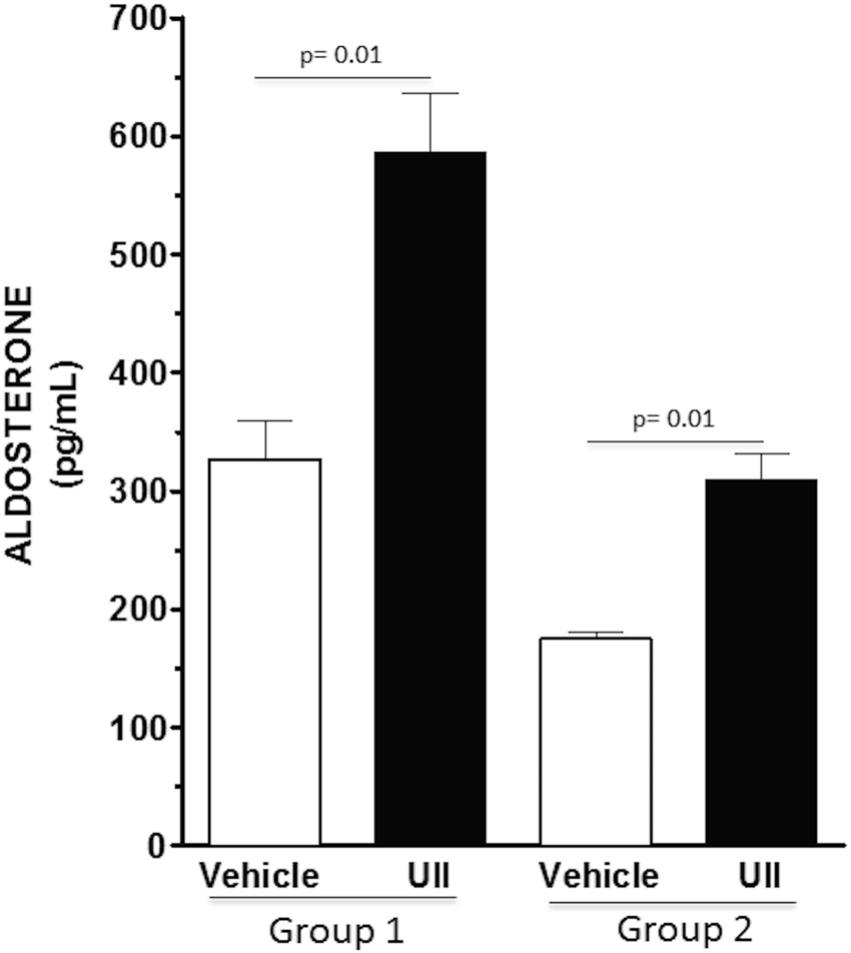
UII infusion induced aldosterone secretion in the zona glomerulosa. UII infusion increased plasma aldosterone concentration (PAC) in rats with normal salt intake and normal renal function (Group 1), and in uni-nephrectomized rats undergoing 2% sodium loading (Group 2). Mean ± SD; n = 5 *per* group.

Plasma aldosterone concentration (PAC) was measured before (day 7) and after the infusion of UII (day 14) via osmotic mini-pumps in Group 1 and Group 2 rats. In Group 1 cases UII infusion markedly increased plasma aldosterone concentration (Fig. 5). In Group 2 controls aldosterone secretion, which was significantly blunted by 47% by the high-salt diet compared to Group 1 controls, was also markedly increased by UII (Fig. 5).

## Discussion

To the best of our knowledge this study is the first to show with radio-telemetry *in vivo* a pressor response to a chronic infusion of UII, which developed after a lag phase of 12-36 hours and then resolved. Due to its transient nature this pressor response was undetectable by measuring BP only at discrete time points after UII administration^21^. Hence, this study resolves the controversy between the pressor effect seen during UII infusion using BP monitoring in acute studies^14–16^, and the lack of detectable pressor effect found in chronic studies where BP was measured with the tail cuff technique^17^.

The transient nature of the pressor effect of UII and its occurrence after a lag phase suggested the activation of endogenous pressor mechanism(s) that were overcome by compensatory phenomena afterward. Of note, this transient pressor response with UII infusion resembled the early BP spike seen in rats after 24 hours of aldosterone infusion^22^^,^^23^, and was reminiscent of the so-called “aldosterone escape phenomenon”^24–26^.

Since UII increased the expression of aldosterone synthase^20^, we focused our mechanistic experiments on the pressor role of UII by investigating renin and aldosterone synthesis activation. To challenge the hypothesis that the transient pressor action of the peptide could be due to enhanced aldosterone secretion two strategies were exploited. By salt-loading the animals and acutely lowering their sodium excretion capabilities with unilateral nephrectomy we found that the escape of BP from UII was abolished, which unambiguously indicates that counteracting sodium excretion transformed the initial BP rise into a sustained and progressive increase of BP. The latter effect was abolished by antagonizing the mineralocorticoid receptor with spironolactone. Accordingly, in salt-loaded uninephrectomized rats the initial and the delayed pressor effects induced by UII involved activation of the mineralocorticoid receptor.

Thus, collectively these findings indicate that: i) UII transiently raises BP via increased endogenous production of aldosterone; ii) when salt intake and sodium excretory capabilities are normal the initial pressor mechanism is counterbalanced by the escape phenomenon; iii) MR antagonism abolishes both the early and late pressor effects of UII.

To clarify if the secretagogue effect of UII only involves a direct action of the peptide on the adrenocortical zona glomerulosa cells, which express functional UII receptor^20^, and/or if renin stimulation is also implicated, we used immunohistochemistry to investigate UII receptor expression in the rat kidney and found this receptor to be detectable in the pre- and post-glomerular arterioles wall. More importantly, we could also show that UII affected renin gene expression (Fig. 3) and that UII interacted differently with salt intake and sodium excretory capabilities in our experimental groups. Notably, UII markedly up-regulated the gene and protein expression of renin while a high sodium intake markedly blunted renin gene expression. Conversely, spironolactone increased it at the mRNA level (Fig. 3), albeit this was not evident at the protein level (Supplemental Table 2), likely because of the small number of samples and the large spread of the values. It should be noted that this up-regulation occurred even in a condition known to suppress renin production, as uninephrectomy and sodium loading. This finding is important for the mechanistic interpretation of the pressor and aldosterone secretagogue effect of UII, and indicates that UII can overcome the renin blunting induced by high sodium intake, both in the presence and in the absence of spironolactone (Fig. 3). Of further note, in Group 4 this effect that was not seen with spironolactone alone, which did not seem to further enhance the activation of renin expression induced by UII.

**Figure 6:**
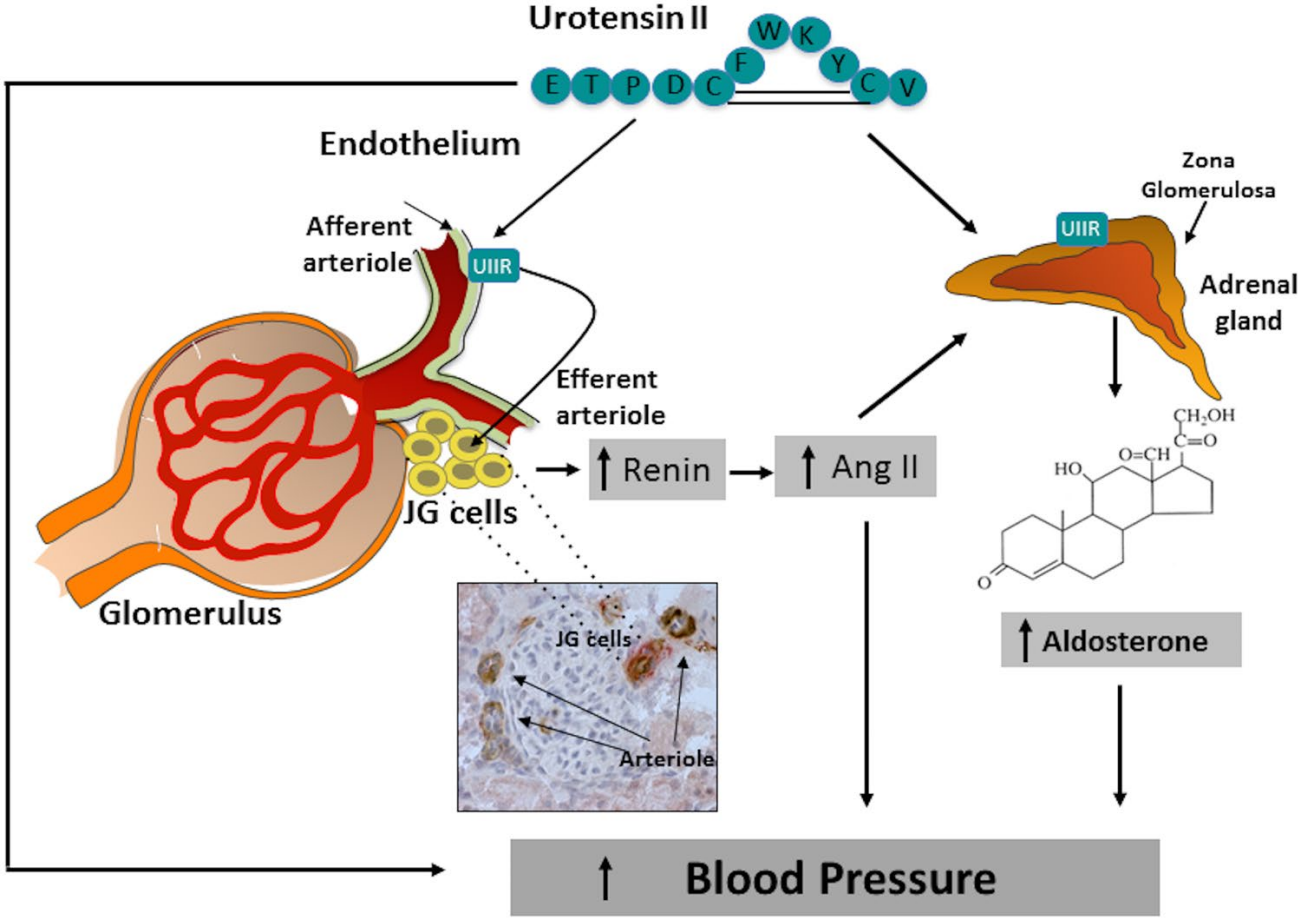
Urotensin II, besides exerting a direct vasoconstrictor effect, enhances the expression of renin in juxtaglomerular (JG) cells and the expression of aldosterone synthase in the adrenocortical zona glomerulosa. Through increased angiotensin (Ang) II formation it further raises the gene expression of aldosterone synthase, thus contributing to increase blood pressure. Under normal salt intake and renal function these pressor effects are counteracted by the escape phenomenon. However, they become evident during salt loading and acutely impaired renal function. **Inset:** Double immunohistochemistry staining shows renin (red) in the juxta-glomerular (JG) cells of the vascular pole of glomeruli and UT-R (brown) in the in endothelium of glomerular arterioles.

Therefore, our study identifies, for the first time, UII as a potent activator of renin expression, suggesting that the peptide might contribute to the secretagogue effect of the peptide on aldosterone through enhanced generation of Ang II (Fig. 6). Overall, these effects can account for the early pressor effect of UII, as well as for the progressive increase of BP during salt-loading in uninephrectomized rats. Of note, multiple findings suggest that elevation of plasma UII levels may be an important background factor in hypertension and cardiovascular complications, particularly in patients with chronic kidney disease and diabetes. The plasma concentrations of UII-like immunoreactivity were 2-fold higher in chronic kidney disease patients not on dialysis, and 3-fold higher in those on haemodialysis (13 ± 3.1 fmol/ml) than in healthy individuals (4.4 ± 1.0 fmol/ml)^6^. Moreover, compared with the latter subjects (4.4 ± 0.02 fmol/ml), they were elevated in diabetes mellitus patients without proteinuria by 1.8-fold (7.8 ± 0.6 fmol/ml) and in those with proteinuria by 1.7-fold (7.3 ± 0.9 fmol/ml), as a result of enhanced UII synthesis in endothelial cells^7^. In this study we choose a dose of UII that previously elicited clear-cut effects on aldosterone synthase in the rat adrenocortical ZG, but we could not measure the levels of UII achieved in plasma of our rats. Hence, even though the effect of renin were evident, we cannot conclusively affirm that our results apply to the aforementioned clinical conditions. Moreover, palosuran, a UII-receptor antagonist that in our hands blunted the increase of CYP11B2 expression in the rat adrenocortical zona glomerulosa^20^, failed to lower BP and microalbuminuria in type 2 diabetic patients, which might argue against the relevance of UII^27^. However, the fact that all those patients were already on multiple antihypertensive agents, including ACE inhibitors or angiotensin receptor antagonists, alongside the UII effects on renin and aldosterone found in this study, can explain those negative results.

With the strength of a randomized design and of radio-telemetry to measure BP, this study showed that urotensin II exerts a pressor effect in normotensive rats with normal renal function and on a normal salt intake, which is transient. In fact it develops after a lag-phase of about 12–36 hours and then resolves swiftly. Due its transient nature this effect could only be evidenced when BP was continuously measured with radio-telemetry, which can reconcile previous conflicting findings.

Under conditions of salt-loading and impaired renal sodium excretory capability, BP progressively increased after the initial pressor effect. Moreover, both early and late pressor responses were abolished by spironolactone indicating that they involve excess activation of the MR. Of importance, our results identify urotensin II as a potent secretagogue of renin, through which it can exert its *in vivo* pressor effect. Not only urotensin II enhanced the expression of renin under normal sodium intake, but also annulled the blunting effect of high sodium intake and unilateral nephrectomy on renin, an effect that can contribute to the progressive increase of BP seen in the groups submitted to these conditions. Of note, the increase of renin induced by UII was paralleled by an increase of aldosterone, which occurred in normotensive rats and also when the secretion of renin was blunted by salt-loading and unilateral nephrectomy (Fig. 5). We further observed only trends, albeit generalized, towards increased renal angiotensin peptide levels in the kidney following UII administration suggesting differences in renin activity (Supplemental Table 2). However, because the number of animals in each group that could be investigated by these experiments was small, and the spread of the data was large, this is a limitation of this study, and further investigation of these changes in a larger number of animals is necessary.

Thus, our results shed light on the pathophysiological role of urotensin II, a peptide known for decades, but whose biological role was obscure thus far. As mentioned, the plasma levels of urotensin II are markedly increased in several clinical conditions, including chronic kidney disease, haemodialysis^6^, and diabetes mellitus^7^, where plasma renin and aldosterone can be inappropriately high for the volume status of the patients. The identification of urotensin II as a potent secretagogue of renin and aldosterone can contribute to accounting for the pressor effect of the peptide when sodium intake is high and/or sodium excretion is impaired.

The discovery of these new actions of urotensin II can open a new avenue of research on the cardiovascular and endocrine effects of this peptide in forms of hypertension, as high-renin primary hypertension, renovascular hypertension, and renin-producing tumors, and chronic kidney disease patients where renin is inappropriately high for prevailing BP and salt intake. At a stage when drug-resistant hypertension is re-emerging potently our results can also provide a background for developing trials on the clinical use of urotensin II receptor antagonists that should consider the effects of urotensin II on renin and aldosterone, at variance with what done so far.

## Methods

All experimental procedures were approved by the Institutional Animal Care and Use Committee and the Italian Ministry of Health and were in accordance with the Guide for the Care and Use of Laboratory Animals, 8^th^ edition, published by the US National Research Council in 2011.

### Experimental groups

Male BP- and weight-matched Sprague-Dawley rats (Charles River Laboratories, Wilmington, MA) at 15 weeks of age were divided into 4 groups of 12 animals each, split into 6 cases and 6 controls (Fig. 1). In Group 1 cases and controls received a 7-days infusion of either UII, or vehicle, respectively, with osmotic mini-pumps (model 2ML1, Alzet, Palo Alto, CA) implanted in the inter-scapular region under general gas anesthesia. This dose of UII (600 pmol/kg/h) was selected based on published data^20^, and on a pilot study that evidenced a pressor effect of UII. In Group 2 the animals receiving the same infusion of UII or vehicle were also submitted to high (2% NaCl) sodium intake at the time of intra-abdominal probe implantation, and to unilateral nephrectomy to antagonize the “escape phenomenon” e.g. the adaptation to hyperaldosteronism by blunting the renal sodium excretory capability. In Group 3, besides nephrectomy and high sodium intake, rats also received the mineralocorticoid receptor (MR) antagonist spironolactone (20 mg/kg/day) that was co-administered with UII. In Group 4 rats received UII and spironolactone infusion.

All animals received the same normal sodium diet; the groups submitted to high sodium intake received 2% NaCl in distilled drinking water to avoid the confounding effects of differences in other nutrients.

All surgical procedures were performed under general anesthesia using a Fluovac Harvard Apparatus (2B Biological Instrument, Varese, Italy) with 4% isoflurane in 100% O_2_ (1 l/min) for induction and 2% isoflurane for maintenance. Adequacy of anesthesia was monitored by pedal response and breathing rate.

On day 14 the rats were anesthetized, weighed, and sacrificed with tiletamine-zolazepam (Zoletil^TM^, 100 mg/Kg) and xylazine (Rompun^TM^, 10 mg/Kg). Organs were removed, weighed and collected for the gene expression and immunohistochemistry studies. The left ventricle (LV) was cleaned from atria and right ventricle and weighed and the LV-to body weight (LV mass index) was calculated to adjust for differences in body weight.

### Radio-telemetry BP measurements and surgical treatment

The Dataquest IV radio-telemetry system (Data Sciences International, Arden Hills, MN) was used for the direct measurement of systolic and diastolic BP. Continuous beat-to-beat recording of BP was performed with the Dataquest IV intra-aortic probe radio-telemetry system (Data Sciences International, Arden Hills, MN, USA). This technique furnishes a large number of readings, thus lowering the spread (SD) of BP values and providing a high statistical power of detecting between-groups differences even with a small number of animals. Implantation of the intra-abdominal aortic BP probes was performed under general gas anesthesia through a midline laparotomy and isolation of the abdominal aorta. After clamping with a silk thread suspension, the aorta was cannulated above bifurcation with a 21 G needle to introduce the tip of the probe inside the aorta. The small entry hole was sealed with 3 M vetbond tissue adhesive (DSI) and a patch of cellulose (DSI). The body of the probe was fixed to the abdominal wall by a suture thread with flexidene. The rats were then placed in individual cages, and left unrestrained and untethered. Systolic and diastolic BP was monitored continuously at a sampling rate of 5 minutes during the 24 hours, starting the day before of UII infusion until the day of sacrifices of the rat. The mean daytime (rest time: 7 AM to 6:59 PM) and night-time values were calculated after identification and exclusion of artifacts and outliers values.

### Renin and Cyp11b2 gene expression

At sacrifice, the left adrenal gland and kidney were snap-frozen in isopentane precooled on dry ice and stored under liquid nitrogen. Total RNA was extracted with the RNAeasy kit (QIAGEN, Milan, Italy) and checked for integrity and quality with a lab-on-chip technology in an Agilent Bioanalyzer 2100 with the RNA6000 Nano assay (Agilent Technologies, Santa Clara, CA). One microgram total RNA was reverse transcribed with Iscript (Bio-Rad) in a final volume of 20 μL. Primers sequences are given in the online Supplemental Table 1.

Real time RT-PCR with Universal Probe Library Probes and Universal Probe Library Assay Design by ProbeFinder Software (Roche, Monza, Italy) in the LightCycler 480 Instrument (Roche, Monza, Italy) was used to measure renin and Cyp11b2 mRNA with the comparative Ct (2^−ΔΔCt^) method as described^28^.

### Aldosterone secretion

The aldosterone concentration in plasma was measured by radioimmunoassays with commercial kits (Aldosterone Maia Kit, Adaltis Italia S.P.A., Bologna, Italy).

### Renin, CYP11B2 and Urotensin II receptor immunohistochemistry (IHC)

At sacrifice the right kidney and adrenal gland were paraformaldehyde-fixed to be used for IHC^29^. The paraffin-embedded tissues were cut in 5 μm-thick sections, dried and then melted at 56 °C for at least 3 hours. After passages through alcohols to remove paraffin, antigen retrieval was performed for 15 min at 121 °C in autoclave followed by treatment with H_2_O_2_ 0.5% for 20 min to inhibit endogenous peroxidases. Kidney tissue sections were incubated with a specific anti-renin antibody (dilution 1:180; Proteintech). After addition of the secondary antibody (goat anti rabbit Immunoglobulin HRP, DAKO), renin was detected with diaminobenzidine.

For double immunnohistochemistry frozen kidney sections were washed with PBS containing 0,2% Tween and incubated overnight with an anti-renin antibody (dilution 1:180; Proteintech) in PBS. Sections were covered with Rabbit AP Polymer; renin was visualized with GBI-Permanent Red solution. Slides were subsequently rinsed thrice in PBS and incubated overnight in PBS containing anti-urotensin II receptor antibody (UT-R, dilution 1:80; Alomone, Jerusalem, Israel). After addition of the secondary antibody (goat anti rabbit Immunoglobulin HRP, DAKO), UT-R was detected with diaminobenzidine.

Adrenal gland sections were blocked with 2% BSA, 0.2% triton and 5% normal goat serum for 1 hour, and then incubated with CYP11B2 antibody (rCYP11B2 1G5-2B2 clone 1:200 dilution) overnight at 4 °C. After washing, they were incubated with secondary antibody (rabbit anti Mouse Immunoglobulin HRP, DAKO) for 1 hour at room temperature and developed using diaminobenzidine. All samples were counterstained with Harris modified hematoxylin (Sigma, Milan, Italy) before mounting.

### Statistical analysis

For descriptive purpose mean and SD, or median and 95% CI, were used, as appropriate. Quantitative data were analyzed after verification of a normal distribution; skewed data underwent transformation to achieve a normal distribution. Comparison across groups was done with one-way ANOVA followed by post-hoc Bonferroni’s test. The BP changes were examined using two-ways repeated measures ANOVA with adjustment for baseline BP values. SPSS 23.00 for Mac (SPSS Italy Inc, Bologna, Italy) and GraphPad 6 for Mac (GraphPad Software, Inc., San Diego, CA) were used for all analyses.

### Data availability statement

The datasets generated during and/or analyzed during the current study are available from the corresponding author on reasonable request.

## Electronic supplementary material

Supplemental materials
