# Supplementary material for: Urotensin II Exerts Pressor Effects By Stimulating Renin And Aldosterone Synthase Gene Expression
Source: Sci Rep. 2017 Oct 24;7:13876. doi: 10.1038/s41598-017-12613-y (PMC5654760; doi:10.1038/s41598-017-12613-y)
Supplement: Supplementary file 1 — Supplemental materials [file 41598_2017_12613_MOESM1_ESM.doc]

**Urotensin II Exerts Pressor Effects**

**By Stimulating Renin And Aldosterone Synthase**

**Gene Expression**

Brasilina Caroccia^#^, Mirko Menegolo^#1^, Teresa M. Seccia, Lucia Petrelli^2^, Michele Antonello^1^, Alice Limena,

Andrea Porzionato^2^, Raffaele De Caro^2^, Marko Poglitsch^3^,

Gian Paolo Rossi*

^#^These authors equally contributed to this work

^1^ Vascular and Endovascular surgery, Department of Cardiac Thoracic and Vascular Sciences,

^2^ Human Anatomy, Department of Molecular Medicine, ^3^Attoquant Diagnostics, Vienna, Austria

and Clinica dell'Ipertensione Arteriosa, Department of Medicine-DIMED,

University of Padova, Italy

___________________________________________________________________

Corresponding author:

Prof. Gian Paolo Rossi, MD. FACC, FAHA.

Clinica medica dell’Ipertensione Arteriosa - DIMED

University Hospital

Via Giustiniani, 2

35126 Padova, Italy

Phone: +39-049-821-2279 or 7821

Fax: +39-49-821-7873

E-mail: [gianpaolo.rossi@unipd.it](mailto:gianpaolo.rossi@unipd.it)

## Renal tissue renin-angiotensin system assay

Angiotensin peptides were measured in the kidney of rats from each group by liquid chromatography tandem-mass spectrometry analysis (LC-MS/MS) at Attoquant Diagnostics GmbH, Vienna, Austria, as described^1^. Kidneys were homogenized in phosphate-buffered saline (PBS) using low-energy sonication.

Renal renin activity was measured by Ang I formation determination in homogenates following recombinant murine angiotensinogen spiking. The enzyme activity is presented as a product formation in ng of the corresponding angiotensin product per μg protein per hour^1^.

Renin activity, Ang 1-10, Ang 1-8, Ang 2-8, Anng 1-7 values in kidney homogenates are reported in Supplemental table 2.

**REFERENCE**

1: Domenig, O. *et al.* Neprilysin is a Mediator of Alternative Renin-Angiotensin-System Activation in the Murine and Human Kidney. *Sci Rep* **6**, 33678 (2016).

**Supplemental Table 1**

| **GENE** | **PRIMER** |
| --- | --- |
| Renin  NM_012642.4 | agacacagccagctttggac  tgatcctggtcatgtctactcc |
| Cyp11b2  NM_012538.1 | gccatcaaagccaactctatg  ttaccaaggggattgctgtc |
| Pbgd  NM_013168.2 | tccctgaaggatgtgcctac  acaagggttttcccgtttg |

**Supplemental table 2: Renal tissue levels of renin and angiotensin peptides (pg/g) and their ratios in the 4 experimental group.**

Data presented as mean ± s.d. NX: nephrectomy; HS 2% diet: high sodium 2% diet; SPIRO: spironolactone.

Please note the consistent increase of renin, Ang 1-10, Ang 1-8, Ang 2-8, Ang 1-7 in Group 2 kidneys of animals exposed to UII as compared to those treated with vehicle. These changes, however, did not attain statistical significance due to the small number of animals. In Group 3 and 4 the increase of renin with UII was smaller, likely because vehicle-treated animals showed higher tissue levels due to spironolactone.

**
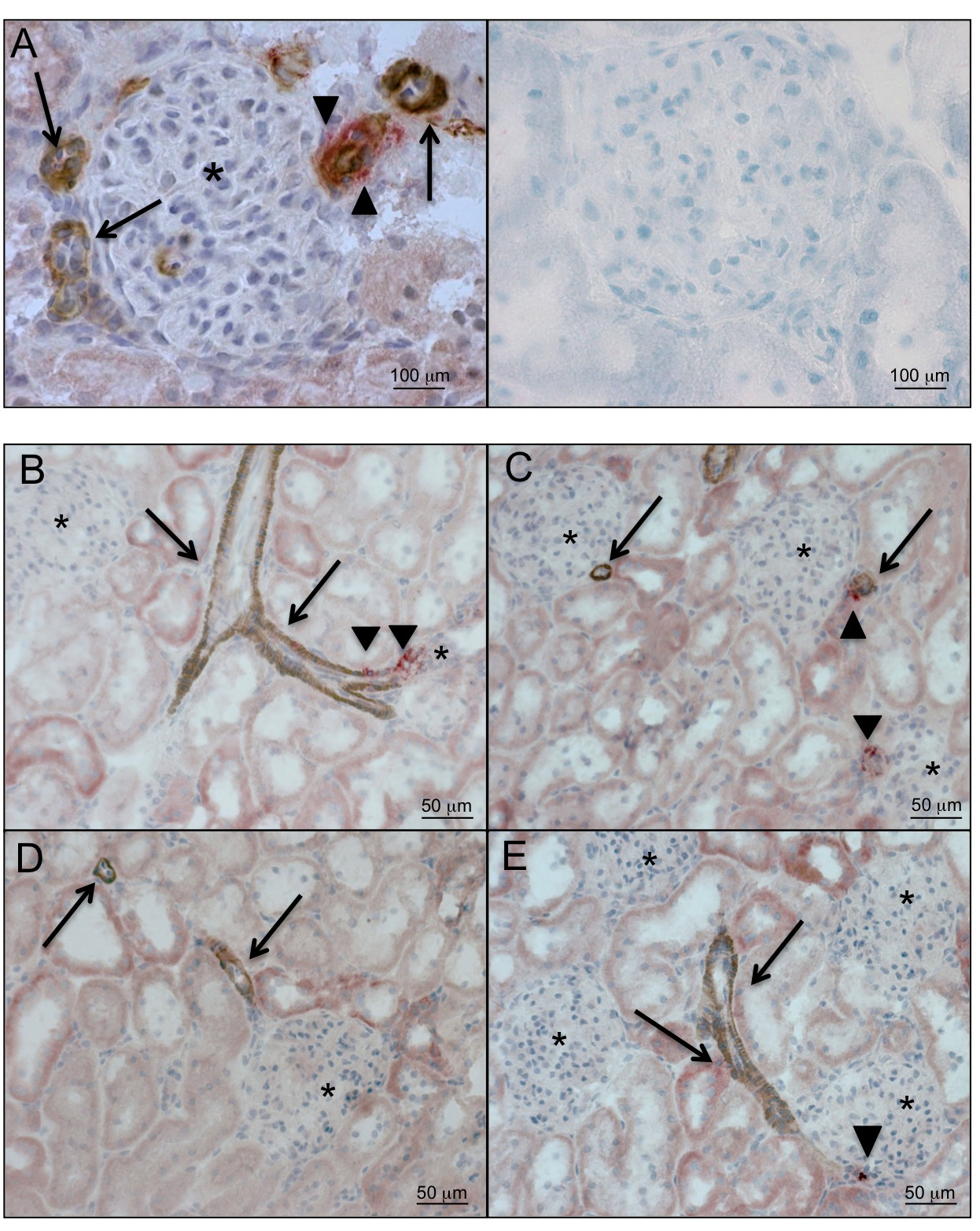
**

**Supplemental Figure 1:** **UT-R expression in pre- and post-glomerular arterioles in the renal cortex and renin expression in juxta-glomerular cells.** Panel A: Representative double immunohistochemistry staining with a specific antibody against the UII receptor (brown) and renin (red) in kidney sections of rats with normal salt intake and renal function. Panel B-C: Immunohistochemical analysis of UII receptor and renin in Group 1 rats (n = 3 rats *per* group, panel B controls, panel C cases). Panel D-E: Immunohistochemical analysis of UII receptor and renin in Group 2 rats (n = 3 rats *per* group, panel D controls, panel E cases). Asterisks mark glomeruli, arrowheads indicate renin, whereas arrow UII receptor. Three rats per group were studied in each group with similar findings.

**
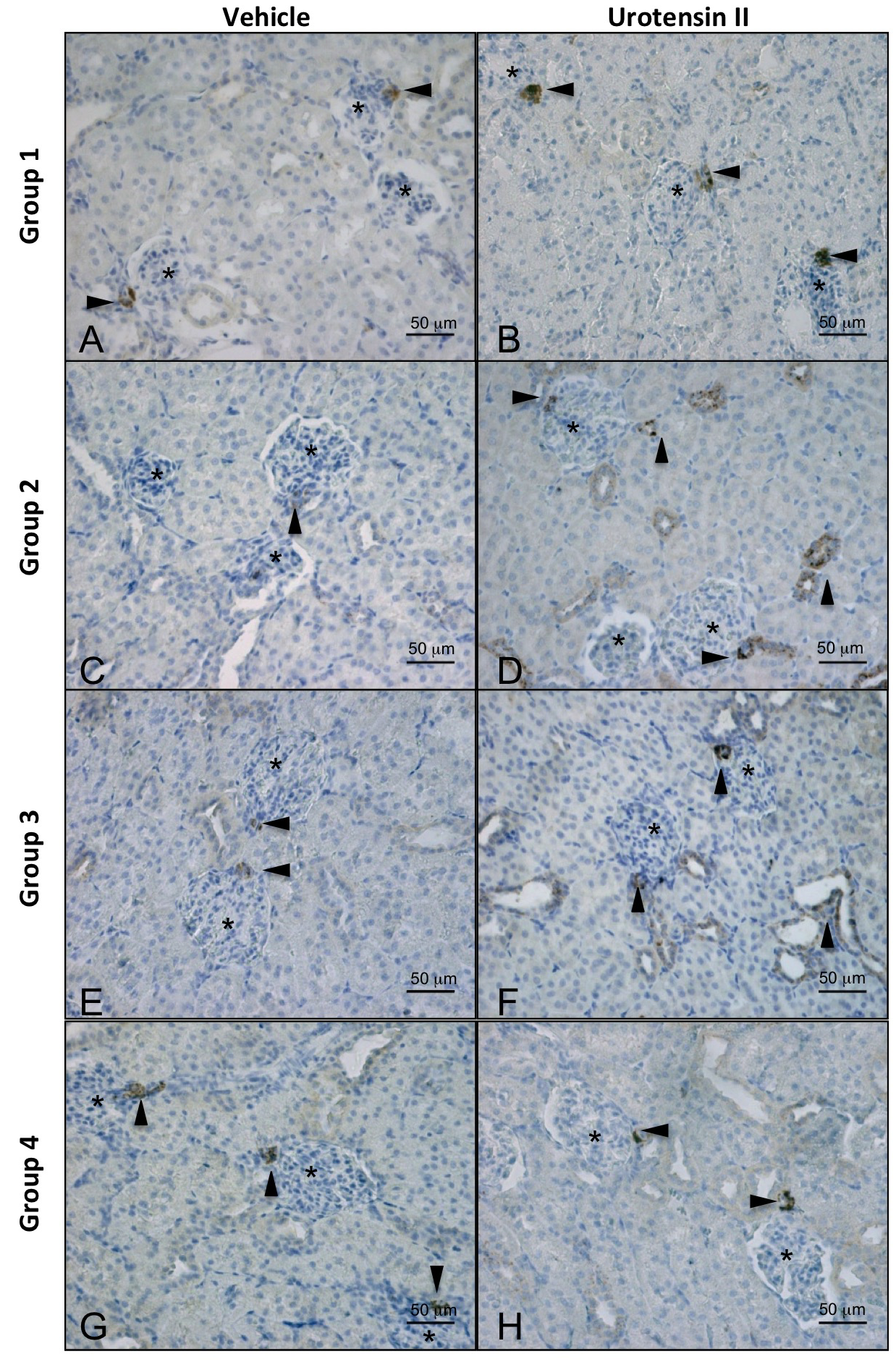
**

**Supplemental Figure 2:** **UII enhanced renin immunostaining in the kidney.** Representative results of immunohistochemical analysis of renin in rats after 1-week infusion of vehicle or UII. Three rats in each group were studied. Asterisks mark glomeruli, arrowheads indicate renin.

**
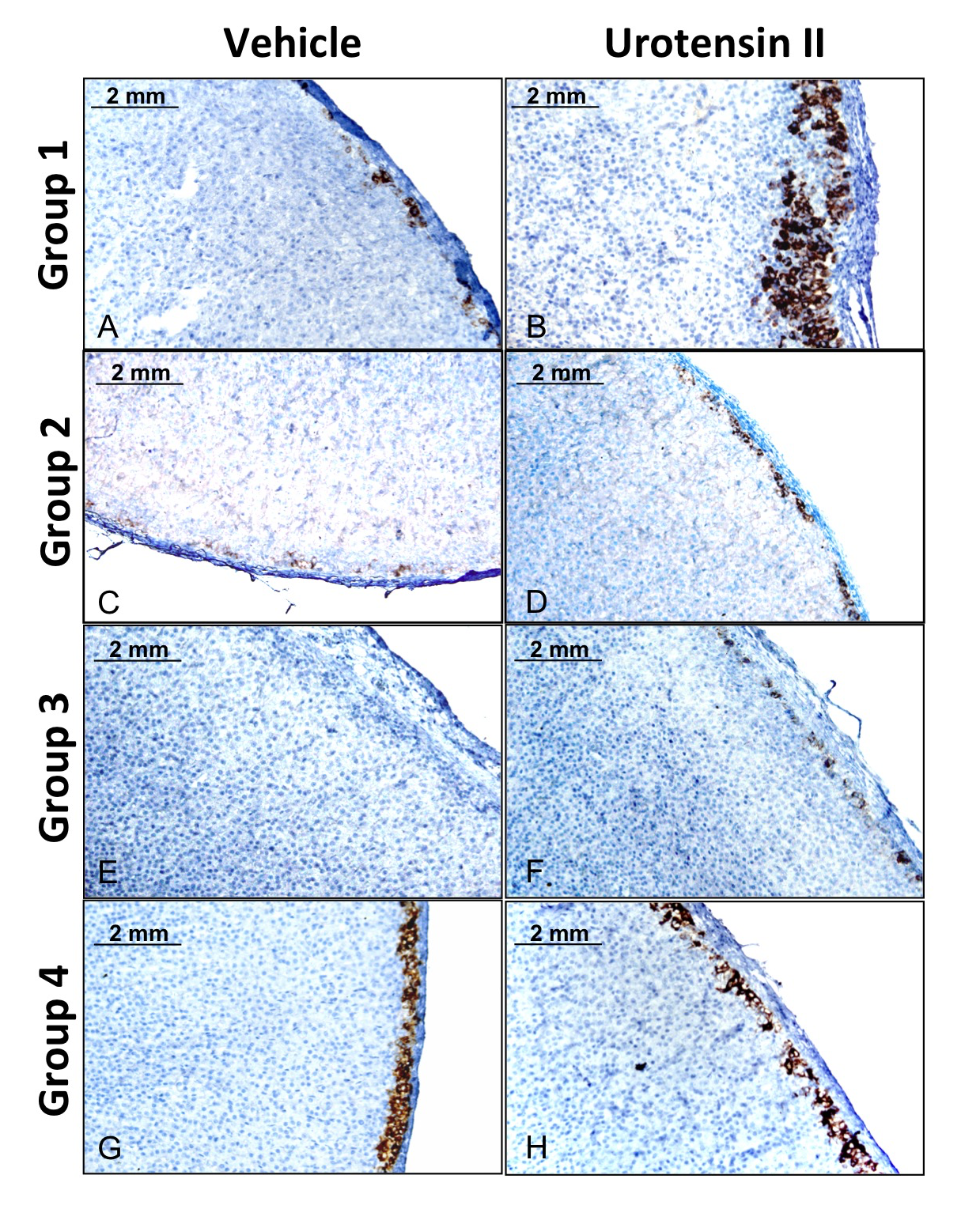
**

**Supplemental Figure 3:** **UII infusion caused a prominent increase in Cyp11b2 immunostaining in the zona glomerulosa.**  Representative immunohistochemistry staining with a specific antibody against CYP11B2. As compared to controls (Panel A), the immunostaining for CYP11B2 of rat adrenocortical zona glomerulosa increased markedly after 1-week infusion of UII (Panel B). Three rats per group were studied in each group with similar findings.
